# Synaptopodin-2 induces assembly of peripheral actin bundles and immature focal adhesions to promote lamellipodia formation and prostate cancer cell migration

**DOI:** 10.18632/oncotarget.3578

**Published:** 2015-03-14

**Authors:** FuiBoon Kai, James P. Fawcett, Roy Duncan

**Affiliations:** ^1^ Department of Microbiology & Immunology, Dalhousie University, Halifax, Nova Scotia, Canada; ^2^ Department of Biochemistry & Molecular Biology, Dalhousie University, Halifax, Nova Scotia, Canada; ^3^ Department of Pediatrics, Dalhousie University, Halifax, Nova Scotia, Canada; ^4^ Department of Pharmacology, Dalhousie University, Halifax, Nova Scotia, Canada; ^5^ Department of Surgery, Dalhousie University, Halifax, Nova Scotia, Canada

**Keywords:** Synpo2, myopodin, membrane protrusions, cancer cell migration, actin cytoskeleton

## Abstract

Synaptopodin-2 (Synpo2), an actin-binding protein and invasive cancer biomarker, induces formation of complex stress fiber networks in the cell body and promotes PC3 prostate cancer cell migration in response to serum stimulation. The role of these actin networks in enhanced cancer cell migration is unknown. Using time-course analysis and live cell imaging of mock- and Synpo2-transduced PC3 cells, we now show that Synpo2 induces assembly of actin fibers near the cell periphery and Arp2/3-dependent lamellipodia formation. Lamellipodia formed in a non-directional manner or repeatedly changed direction, explaining the enhanced chemokinetic activity of PC3 cells in response to serum stimulation. Myosin contraction promotes retrograde flow of the Synpo2-associated actin filaments at the leading edge and their merger with actin networks in the cell body. Enhanced PC3 cell migration correlates with Synpo2-induced formation of lamellipodia and immature focal adhesions (FAs), but is not dependent on myosin contraction or FA maturation. The previously reported correlation between Synpo2-induced stress fiber assembly and enhanced PC3 cell migration therefore reflects the role of Synpo2 as a newly identified regulator of actin bundle formation and nascent FA assembly near the leading cell edge.

## INTRODUCTION

The podins are a unique family of proline-rich, actin binding proteins that play important roles in several normal and pathologic cell and tissue processes. The founding member of the family, synaptopodin-1 (Synpo1), promotes formation of kidney podocytes and the dendritic spine apparatus of telencephalic synapses [1, 2]. These functions have been linked to Synpo1 alteration of actin dynamics and cell motility; Synpo1 regulates filopodia formation [3], α-actinin actin bundling activity [4], and RhoA signaling and stress fiber (SF) biogenesis [2, 5]. Synaptopodin-2 (Synpo2), also known as myopodin, is the second identified member of the podin family [6]. Analysis of invasive versus indolent prostate cancer tissues revealed Synpo2 expression, which is predominant in prostate acinar epithelial and basal cells, was dramatically reduced in >92% of invasive prostate cancer tissues, and loss of Synpo2 expression correlates with prostate cancer relapse [7, 8]. Loss of Synpo2 expression due to methylation-dependent epigenetic silencing of gene expression is also associated with invasive bladder cancer [9, 10], suggesting Synpo2 is a repressor of tumor cell invasion.

The relationship between Synpo2 expression and tumor development is unclear. Several studies, based on ectopic expression of Synpo2 in prostate cancer PC3 cells, an invasive cell type with very low levels of Synpo2 protein expression due to a hemizygous deletion [11], indicate Synpo2 suppresses cell invasion *in vitro*, and inhibits tumor development and metastasis *in vivo* [11, 12]. It is unclear whether these effects are due to Synpo2 inhibition of PC3 cell migration: ectopic Synpo2 expression has been reported to decrease or have no effect on PC3 cell migration [11-13], while siRNA-mediated inhibition of Synpo2 expression reduces PC3 cell migration [14] and ectopic expression increases collagen invasion of HEK293T cells and mouse myoblasts [15]. We recently demonstrated that Synpo2 alters the RhoA/ROCK signaling response of PC3 cells to external migration stimuli, and can either increase or decrease cell motility depending on the stimulus [16]. This potentially important invasive cancer biomarker therefore exerts complex effects on the cellular response to external migration stimuli. As such, loss of Synpo2 expression could reflect increased migration of neoplastic prostate epithelial cells, or decreased migration and interaction of basal cells leading to loss of integrity of the basal layer.

Previous studies provide some insights into how Synpo2 could affect cancer cell migration responses. We recently determined that all five isoforms of Synpo2 induce formation of, and co-localize with, morphologically and biochemically distinct ventral SFs in the cell body of PC3 cells following serum stimulation [17]. These results are consistent with our previous demonstration that Synpo2 activates RhoA [16], a key regulator of SF formation [18]. Inhibiting Synpo2-induced SF assembly also prevents Synpo2-enhanced prostate cancer cell migration in response to serum-stimulation [17], indicating a direct correlation between SF assembly and a Synpo2 pro-migratory phenotype. In addition, Synpo2 homologues from various species enhance actin nucleation, polymerization and bundling *in vitro* [19, 20], and Synpo2 has been shown to interact with focal adhesions (FAs) and FA-associated proteins [12, 13, 21]. These studies suggest Synpo2 is a potentially important regulator of actin dynamics and FA assembly. However, the relationship between cell migration responses and Synpo2 effects on actin or FA dynamics in prostate cancer cells are unclear.

During cell migration, actin polymerization and FA assembly at the leading edge drives formation of membrane protrusions. Lamellipodia are sheet-like, Arp2/3 complex-dependent membrane protrusions ~1-2 μm thick that contain a dense network of branched actin filaments [22, 23]. Filopodia contain fascin-crosslinked linear actin filaments embedded in or protruding from lamellipodia [24]. Development of these actin structures stimulates formation of nascent FAs that serve as molecular clutches, reducing retrograde F-actin flow and promoting advancement of the leading edge [25-28]. While non-muscle myosin II (NM II) is not required for nascent FA formation, maturation of early FAs into elongated, stable FAs is dependent on tension exerted by NM II in the lamellum, an ~2-5 μm thick region of bundled actin networks immediately behind lamellipodia [29]. Recent studies support a model whereby myosin contraction also drives integration of F-actin structures at the leading edge into stress fibers in the cell body, resulting in cell body translocation, tail retraction and cell advance [27, 30-33]. The full complement of actin regulators involved in this process are not yet well established.

Using live cell imaging and immunofluorescence microscopy, we now show that Synpo2 dramatically increases formation of Arp2/3-dependent membrane protrusions in response to serum stimulation. Results further indicate Synpo2 stimulates cell migration by promoting formation of nascent FAs and actin bundles at the leading cell edge, and these Synpo2-associated actin bindles flow centripetally to generate SFs in the cell body. Thus, Synpo2 affects cell motility by functioning as a new positive regulator of membrane protrusions and FA assembly.

## RESULTS

### Synpo2 promotes random PC3 cell migration and Arp2/3-dependent lamellipodia formation

The majority of studies on Synpo2 in the context of invasive cancer cell migration use PC3 prostate cancer cells and ectopic expression of the Synpo2As isoform, a 698-residue isoform also referred to as ΔN-MYO1 [17]. Using this system, it was determined that conditioned medium from NIH 3T3 cells is a potent stimulator of PC3 cell motility, and ectopic expression of Synpo2 partially inhibits (~20-40%) this migratory response to variable extents [11, 13, 16]. In contrast, FBS provides only a weak migration stimulus to PC3 cells, but Synpo2 expression dramatically upregulates this response, increasing cell migration by 3-5-fold [14, 16]. In view of the above considerations, we used retrovirus transduction to stably express Synpo2As (hereinafter referred to as Synpo2) in PC3 cells, and compared the effects of serum stimulation of vector-transduced versus Synpo2-transduced PC3 cells on actin and FA dynamics.

**Figure 1:**
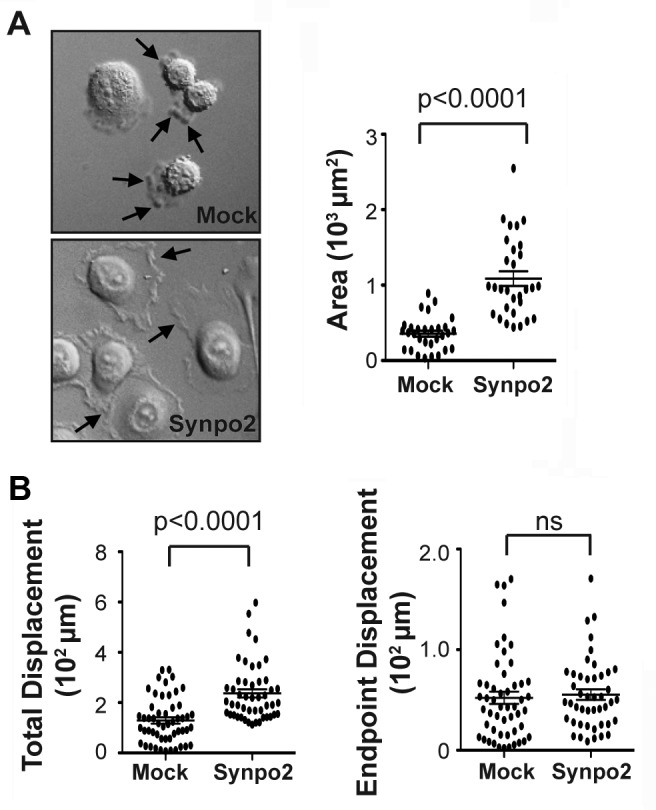
Synpo2 promotes random membrane protrusions and non-directional cell migration (A) Morphological changes in serum synchronized mock- and Synpo2-transduced PC3 cells were imaged using DIC microscopy (left panel), and the mean area surrounding nuclei ± SEM for 30 cells was determined using ImageJ (right panel). Arrows in top image (left panel) indicate membrane blebs, and those in the bottom image lamellipodia-like membrane protrusions. Images are stills from Supplementary Video 1. (B) Total displacement (left panel) and endpoint displacement (right panel) were calculated for 45 cells. Horizontal bars in all graphs are the mean ± SEM.

To confirm and extend previous migration results based on transwell migration assays, we used live cell imaging to assess migration of mock- and Synpo2-transduced PC3 cells cultured in the presence of serum. Video microscopy revealed PC3 cells expressing Synpo2 were more motile than mock-transduced cells (Supplementary Video 1). Synpo2-transduced cells displayed highly dynamic membrane protrusions that differed from those observed in mock-transduced cells. Mock-transduced cells predominantly exhibited a rounded cell morphology with extensive membrane blebbing, while Synpo2-expressing cells displayed diminished blebbing or rapid interconversion from a blebbing phenotype to one with very large and dynamic sheet-like membrane protrusions (Figure 1A and Supplementary Video 1). Following serum-starvation, by 30 min post-serum stimulation these membrane protrusions increased the average cell area of migrating Synpo2-expressing cells >2-fold relative to mock-transduced cells (Figure 1A). The protrusions formed in a non-directional manner, or repeatedly changed their orientation as cells established a new leading edge and switched direction (Supplementary Video 1). Tracking the migration paths of individual cells at 1.5-min intervals for 2.5 h (Supplementary Figure 1A) indicated the total path travelled by Synpo2-expressing cells was on average two-fold higher than that of mock-transduced cells, while the endpoint displacement values (i.e., the shortest distance between the starting point to the end point) were indistinguishable (Figure 1B). Thus, the recently reported ability of Synpo2 to increase chemokinetic PC3 cell migration in response to serum stimulation [16] reflects the effects of Synpo2 on promoting extensive, random formation of membrane protrusions.

### Synpo2 promotes Arp2/3-dependent lamellipodia formation in PC3 cells

**Figure 2:**
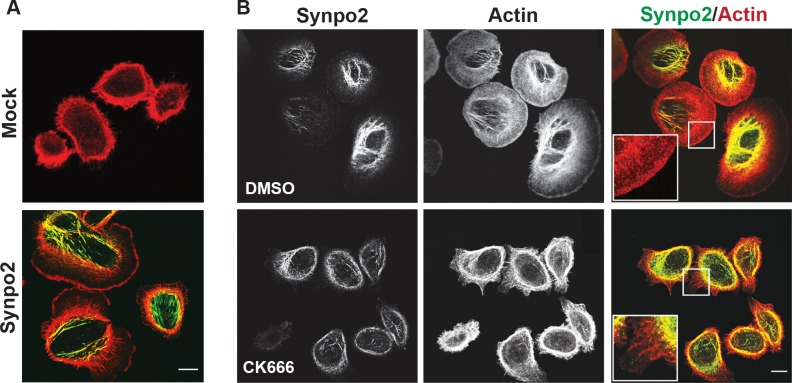
Synpo2 promotes Arp2/3-dependent lamellipodia formation (A) Following 24 h of serum-starvation, mock- or myc-tagged Synpo2-transduced PC3 cells were stimulated with serum-containing growth medium for 30 min prior to being stained with phalloidin (red) to detect F-actin and immunostaining with anti-myc antibody (green). (B) Myc-tagged Synpo2-transduced PC3 cells were serum stimulated for 30 min in the absence (DMSO) or presence of the Arp2/3 inhibitor CK666, then immunostained with anti-myc antibody (green) and phalloidin (red). Boxed areas are magnified 250% in the insets. Scale bar=10 μm.

Phalloidin staining of filamentous actin (F-actin) indicated the sheet-like protrusions generated in Synpo2-expressing cells contained actin-rich rims ~1 micrometer in diameter (Figure 2A), a characteristic feature of lamellipodia [30], and Synpo2 predominantly colocalized with the induced SFs that formed in the cell body, as recently reported [17]. These actin-rich membrane protrusions and SFs were not evident in serum-stimulated, mock-transduced cells (Figure 2A). Generation of a dendritic actin network near the leading edge promotes lamellipodia formation, a process that involves stimulation of branched actin polymerization by the Arp2/3 actin nucleation complex [34]. To determine whether the Synpo2-induced sheet-like protrusions are lamellipodia, cells were treated with CK666, a highly specific inhibitor of the Arp2/3 complex [35, 36]. DMSO-treated cells contained the large, actin-rich, sheet-like membrane protrusions and extensive Synpo2-associated actin SFs in the cell body (Figure 2B). In contrast, Synpo2-expressing cells treated with CK666 were dramatically reduced in formation of the large membrane protrusions; cells instead displayed an F-actin network around the cell periphery that contained numerous parallel actin fibers perpendicular to the membrane (Figure 2B). Based on morphology, actin staining pattern and Arp2/3-dependence, we conclude that Synpo2 promotes lamellipodia formation in serum-stimulated PC3 cells, and that the chemokinetic migration phenotype promoted by Synpo2 reflects continuous directional changes in lamellipodia formation.

### Myosin contraction promotes retrograde flow of Synpo2-induced actin bundles at the cell edge and stress fiber formation in the cell body

**Figure 3:**
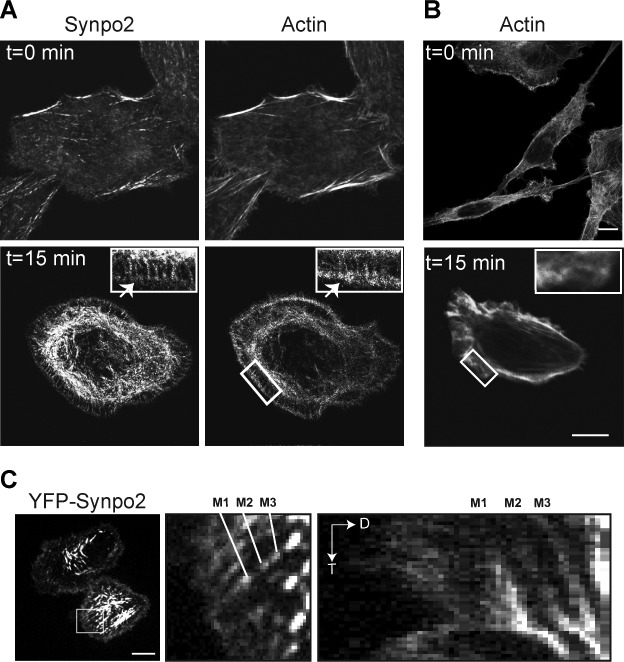
Synpo2 flows centripetally under chemokinetic conditions (A) Myc-tagged Synpo2-expressing PC3 cells were serum-synchronized and stained with phalloidin to detect F-actin and immunostained with anti-myc antibody at t=0 or t=15 min post-serum stimulation. Arrow in the insets indicates Synpo2 colocalized with F-actin within the actin-rich edge of the lamellipodium. Boxed areas are magnified 250% in the insets. (B) As in panel A using phalloidin staining of mock-transduced cells to show radial SFs are not induced by serum stimulation. (C) Serum-synchronized PC3 cells transfected with YFP-tagged Synpo2 were imaged using live cell fluorescence microscopy. Left panel is an image at 30 min post-serum stimulation. Boxed area is magnified 533% in the middle panel. Right panel is a kymograph (distance [D] versus time [T]) showing centripetal movement (indicated by the downward and rightward trajectory) of the three Synpo2 puncta highlighted in the middle panel (M1-3). Scale bars=10 μm.

Additional studies were performed to further investigate the effects of Synpo2 on actin dynamics in PC3 cells. In the absence of serum-stimulation, Synpo2 stained diffusely throughout the cell and partially colocalized with F-actin at the cell periphery (Figure 3A; top panels). Within 15 min post-serum stimulation, Synpo2-expressing cells generated actin-rich membrane protrusions and complex networks of Synpo2-associated actin bundles in the cell body and in the lamellum, the actin-rich region immediately behind the lamellipodium [28] (Figure 3A; bottom panels). Numerous actin fibers oriented perpendicular to the cell edge also appeared in Synpo2-expressing cells, and Synpo2 partially colocalized with these fibers (Figure 3A; insets in bottom panels). These radial actin fibers were not observed in serum-stimulated mock-transduced cells (Figure 3B). One end of these Synpo2-induced radial fibers terminated within the actin-rich rim of the forming lamellipodia while the other end merged with the Synpo2-rich actin bundles forming in the lamellum. This arrangement of Synpo2-induced actin fibers in PC3 cells resembles the relationship between dorsal or radial SFs and transverse arcs noted in other cell types [33].

Retrograde flow of actin fibers from the cell periphery is known to contribute to formation of contractile SFs in the cell body [32, 33]. It therefore seemed likely that formation of Synpo2-induced SFs in the cell body might reflect this newly described function of Synpo2 as a promoter of actin bundle formation at the leading cell edge. To confirm this prediction, the dynamics of YFP-tagged Synpo2 in PC3 cells was examined by live cell imaging. Synpo2 at the cell periphery was highly dynamic, oscillating in waves of protrusion and retraction (Supplementary Video 2, left panel). Time-lapse composite images (Supplementary Video 2, right panel) indicated Synpo2 moved centripetally and became incorporated into Synpo2-associated SFs in the cell body. Kymograph analysis confirmed the centripetal flow of Synpo2, as evident from the time-dependent inward trajectory of Synpo2 from the cell periphery (Figure 3C). To examine retrograde flow of both Synpo2 and actin, stably transduced PC3 cells expressing RFP-tagged Lifeact, an actin-binding peptide used to visualize F-actin [37], were co-transfected with GFP-tagged Synpo2 and observed by live cell imaging. Cells not exhibiting detectible Synpo2 expression (Supplementary Video 3) exhibited no obvious inward flow of F-actin. In contrast, Synpo2-associated actin bundles near the cell periphery were observed flowing centripetally from the periphery to developing actin bundles in the cell body (Supplementary Video 3).

**Figure 4:**
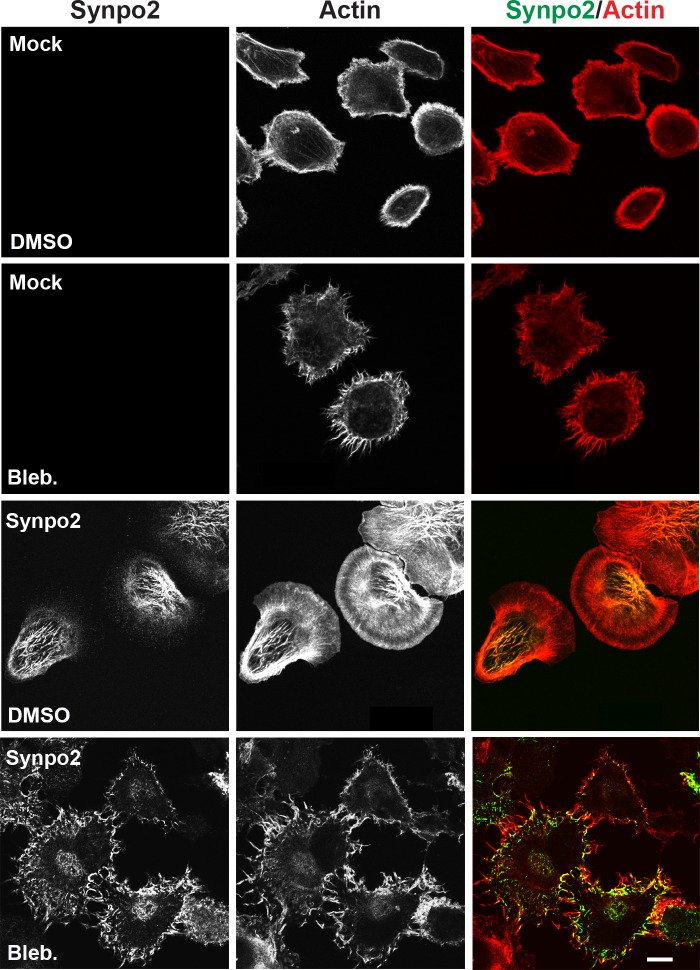
Formation of Synpo2-induced stress fibers in the cell body requires myosin contraction Mock- and Synpo2-transduced PC3 cells were starved overnight followed by FBS stimulation in the presence of 75 μM blebbistatin for 30 min to inhibit NM II contraction prior to staining with phalloidin (red) to detect F-actin and immunostaining with anti-myc (green) antibodies to detect Synpo2. Scale bar=10 μm.

Myosin contraction is known to promote incorporation of actin bundles near the cell periphery into SFs in the cell body [38]. In both mock- and Synpo2-transduced PC3 cells, inhibiting NM II contraction using a high dose (75 μM) of blebbistatin resulted in extensive formation of actin-rich, filopodia-like membrane protrusions (Figure 4), a phenotype noted in other cell types treated with blebbistatin [39]. Most notably, inhibiting NM II dramatically reduced formation of the complex Synpo2-associated SF network in the cell body of Synpo2-expressing cells. Instead, Synpo2 stained diffusely through the lamellar region and further into the cell body, with intense staining near the cell periphery, particularly near the base of the filopodia-like actin-rich protrusions (Figure 4). Synpo2 therefore induces actin fiber assembly at the cell periphery, with NM II-dependent retrograde flow of Synpo2-associated actin fibers from the cell edge contributing to SF formation in the cell body.

### Synpo2-induced membrane protrusions and enhanced PC3 cell migration are independent of NM II contraction and FA maturation

**Figure 5:**
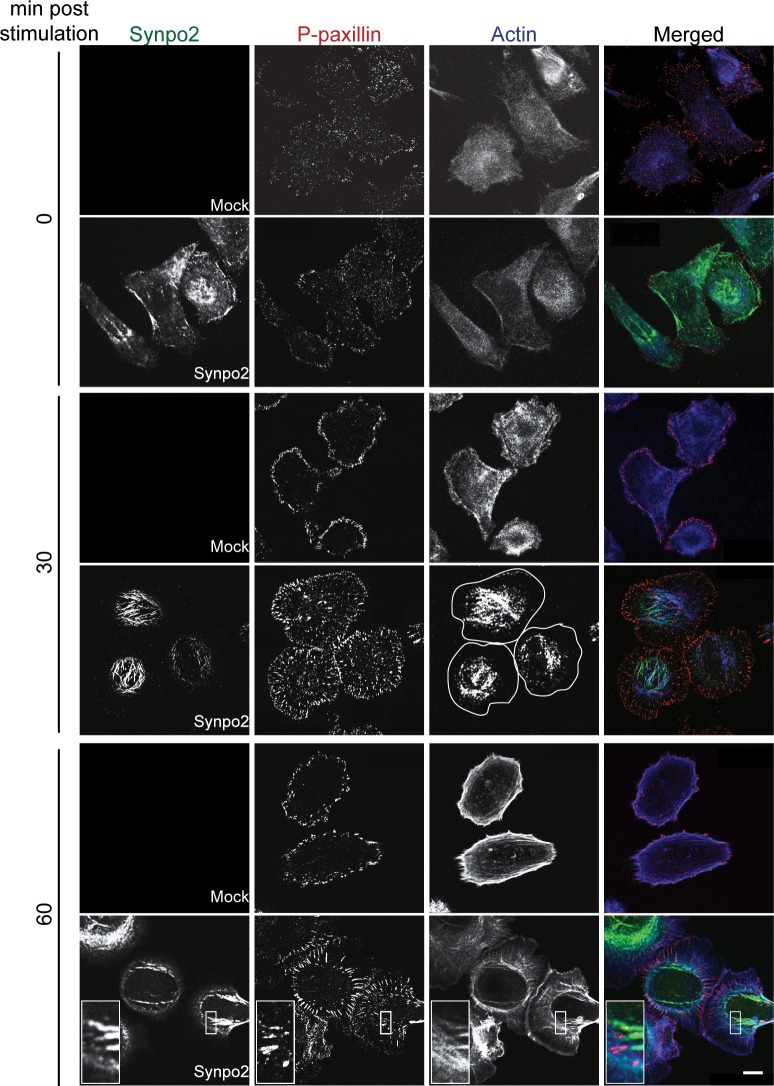
Focal adhesion maturation in Synpo2-expressing PC3 cells Serum-synchronized mock- and myc-tagged Synpo2-expressing PC3 cells were immunostained at the indicated times with anti-myc (green) and anti-phospho-paxillin (red) antibodies, and with phalloidin (blue) to detect F-actin. The boxed area is magnified 375% in the insets (bottom row) to show the ends of Synpo2-induced actin bundles in proximity to paxillin-containing focal adhesions. Cell peripheries are outlined in white in the Synpo2/30 min/actin panel. Scale bar=10 μm.

Formation of membrane protrusions at the leading cell edge is dependent on formation and maturation of FAs [25], and Synpo2 is a component of the FA proteome and interacts with FA-associated proteins [12, 13, 21]. To investigate whether Synpo2 promotes lamellipodia formation via effects on FA dynamics, serum-stimulated cells were immunostained for phospho-paxillin, an early component of nascent FAs [40]. Early, immature FAs (also known as nascent adhesions and/or focal complexes) in serum-starved mock- and Synpo2-transduced cells appeared as small dots throughout cells (Figure 5; t=0 min). By 30 min post-serum stimulation, Synpo2-expressing cells developed numerous centripetally arranged, larger FAs throughout cells while FAs in serum-stimulated mock-transduced cells maintained a dot-like staining pattern concentrated near the cell periphery. Within 1 h of serum stimulation, nascent FAs in Synpo2-expressing cells developed into elongated, centripetally-oriented mature FAs closely associated with actin bundles in the lamellum and the cell body (Figure 5; insets). Similar to earlier timepoints, FAs in mock-transduced cells remained undeveloped and concentrated at the cell periphery. Quantitatively, Synpo2-expressing cells had a 4-fold increase in total FAs and an 8-fold increase in mature FAs (larger than 1 μm^2^) relative to mock-transduced cells (Supplementary Figure 1B).

**Figure 6:**
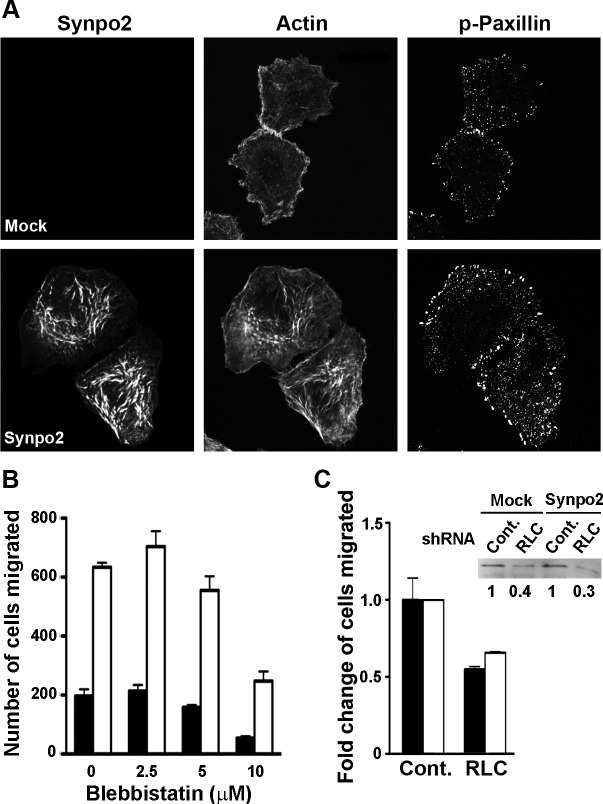
Synpo2-enhanced lamellipodia formation and cell migration is independent of myosin and focal adhesion maturation (A) Mock- and myc-tagged Synpo2-transduced PC3 cells were serum-synchronized in the presence of 10 μM blebbistatin for 1 h, then immunostained with anti-myc and anti-phospho-paxillin antibodies, and with phalloidin. Scale bar=10 μm. (B) Transwell migration assays of mock- and Synpo2-transduced PC3 cells in the presence of serum stimulation and the indicated doses of blebbistatin. Results are reported as the mean number of cells that transmigrated ± SEM from three independent experiments conducted in duplicate. (C) Effect of shRNA myosin RLC knockdown on mock- and Synpo2-transduced PC3 cell migration was assessed using transwell migration assays. Results are presented as mean fold change in cells migrated of RLC- relative to control-knockdown cells ± SEM from three independent experiments conducted in triplicate. The fold changes in RLC expression of stably transduced PC3 cells expressing a non-targeting shRNA (Cont.) or RLC-targeted shRNA, and co-transduced with empty vector (Mock) or Synpo2-expressing vector were quantified by western blotting with anti-RLC antibody (inset).

FA maturation is dependent on tension generated by NM II motor activity [26, 29], and the rabbit and chicken homologues of Synpo2 bind smooth muscle myosin [41], suggesting Synpo2 might promote protrusion formation via direct effects on FA maturation. Inhibition of NM II activity was used to determine whether FA maturation is the cause or an effect of Synpo2-induced membrane protrusions. Mock- and Synpo2-transduced cells were serum-stimulated in the presence of a low dose (10 μM) of the NM II ATPase inhibitor blebbistatin, and cells were imaged at 1 h post-serum stimulation, a time when FA maturation and membrane protrusion was pronounced in Synpo2-expressing cells. Low-dose blebbistatin treatment only partially impairs SF formation [42], and Synpo2-expressing cells still contained complex networks of Synpo2-associated SFs in the cell body (Figure 6A). While larger FAs appeared more numerous within the lamellipodium and the lamellum of Synpo2-expressing cells compared to mock-transduced cells following blebbistatin treatment, the large elongated mature FAs that appeared by 60 min in untreated cells (Figure 5) were not apparent in blebbistatin-treated Synpo2-expressing cells (Figure 6A). Although FA maturation was evidently impaired, Synpo2-expressing cells still displayed large lamellipodia.

Furthermore, transwell migration assays indicated blebbistatin treatment did not inhibit the ability of Synpo2 to promote PC3 cell migration in response to serum stimulation. Increasing doses of blebbistatin inhibited the migration of both mock- and Synpo2-transduced cells to the same degree (Figure 6B), implying NM II ATPase activity is required for inherent PC3 cell motility. However, blebbistatin did not affect the ~3-fold difference in migration of mock- versus Synpo2-transduced cells (Figure 6B). The blebbistatin results were confirmed using mock- or Synpo2-transduced PC3 cells stably expressing non-targeting control or myosin regulatory light chain (RLC)-targeted shRNAs. A 60-70% decrease in NM II RLC expression decreased the inherent motility of PC3 cells in transwell migration assays, but had no effect on Synpo2-enhanced cell migration (Figure 6C). Thus, Synpo2-induced membrane protrusion formation and enhanced cell migration are both independent of NM II contraction and tension-dependent FA maturation.

## DISCUSSION

Synpo2 is the second identified member of the podin family of actin binding proteins, and is associated with invasive tumor development. This interesting family of actin regulators affects Rho signaling pathways, actin cytoskeleton dynamics and cell motility [43], although the relationships between these different processes are still poorly understood. Recent results revealed Synpo2 induces formation of ventral SFs in the cell body, the generation of which directly correlates with enhanced PC3 cell migration [17]. We now show that Synpo2-induced SF formation in the cell body is an incidental effect of retrograde F-actin flow from the cell periphery, and that SF formation reflects the role of Synpo2 as a newly identified effector of actin bundle and lamellipodia formation at the leading cell edge.

Recent results indicated Synpo2 increases the chemokinetic activity of serum-stimulated PC3 cells [16], a property that correlates with Synpo2-induced SF formation in the cell body [17]. The present results provide mechanistic explanations for both of these Synpo2 phenotypes. As we now show, enhanced non-directional cell migration is attributable to Synpo2-induced non-directional lamellipodia formation that results in increased total cell displacement but no change in endpoint cell displacement (Figure 1 and Supplementary Video 1). Live cell imaging also revealed retrograde flow of Synpo2-associated actin bundles from the cell periphery and their incorporation into SFs in the cell body (Figure 3 and Supplementary Videos 2 and 3), and inhibiting NM II motor activity abrogated SF formation but did not inhibit Synpo2-induced formation of lamellipodia or enhanced cell migration (Figures 4 and 6). SF formation based on retrograde flow of actin filaments has been described in other systems [30-32, 44, 45]. Together, these results indicate SF formation is a consequence of Synpo2 effects on actin dynamics and membrane protrusion at the cell periphery, with the latter events contributing to enhanced prostate cancer cell migration in response to serum stimulation.

These newly described functions of Synpo2 contrast markedly with the known functions of Synpo1, the founding member of the podin family. Both Synpo1 and Synpo2 induce RhoA-dependent SF formation. However, Synpo2-induced SFs are associated with increased, non-directional cell migration while Synpo1-induced SF assembly favors a contractile, rather than a motile, cell phenotype or promotes directed migration [2, 5]. Similar dichotomies exist in the mechanisms employed by the podins to regulate RhoA pathways. Synpo1 inhibits proteasomal degradation of RhoA while Synpo2 increases levels of activated RhoA-GTP [2, 16]. The precise role of RhoA activation in podin-enhanced cell migration is unclear. NM II phosphorylation by the RhoA effector kinase ROCK promotes SF fiber formation, tail retraction and cell body translocation [46]. This may explain how Synpo1 affects cell migration, and might also explain decreased inherent PC3 cell motility when NM II was inhibited (Figure 6). However, this is not the mechanism responsible for Synpo2-stimulated formation of actin-rich membrane protrusions and cell migration, both of which are NM II-independent. One possibility is Synpo2 alters the relative levels of different activated Rho GTPases. For example, modest increases in the level of activated Rac increases non-polarized lamellipodia formation and promotes a switch from directionally persistent to random migration [47], similar to what occurs in Synpo2 expressing cells. However, inhibiting Rac activity does not alter the Synpo2-induced enhanced migration phenotype [16]. Since RhoA, Rac1 and Cdc42 are all activated at the leading cell edge [48], additional studies are needed to determine whether modest spatiotemporal changes in Rho GTPase activation contribute to the random migration property of Synpo2-expressing cells.

Formation of immature FAs and membrane protrusions correlated with the activity of Synpo2 as an effector of actin bundle formation at the cell edge. Previous *in vitro* studies indicated the rabbit and chicken homologues of Synpo2 possess actin nucleation, polymerization and bundling activity [19, 20, 41]. The present results provide a biologically relevant context for these *in vitro* actin regulatory functions of human Synpo2. A recent report revealed purified chicken Synpo2 can initiate actin polymerization and generate linear actin filaments that assemble into highly ordered, parallel actin bundles [49]. It therefore seems plausible that Synpo2 mediates actin polymerization to generate parallel actin filaments at the leading edge; such filaments were evident in Synpo2-expressing cells shortly after serum-stimulation (Figure 3), and in cells where Arp2/3 actin branching activity or NM II contraction were inhibited (Figures 2 and 4). Generation of these filaments also provides an explanation for increased formation of immature FAs in blebbistatin-treated Synpo2-expressing cells (Figure 6), since actin polymerization at the leading edge promotes nascent FA assembly in the absence of NM II contraction [26]. We therefore suggest that Synpo2 upregulation of actin bundles and nascent FAs near the cell periphery provide sufficient traction points for Synpo2-enhanced membrane protrusion and the enhanced migration phenotype. Additional actin regulatory proteins may also be involved in this process. For example, Synpo1 and 2 both bind to α-actinin [4, 17, 41, 50], and Synpo1 promotes bundling and elongation of α-actinin-induced actin filaments in cells, converting short branched filaments into long unbranched filaments [4]. Similar interactions between Synpo2 and α-actinin, or with other actin regulatory proteins, might well be functionally relevant to Synpo2 effects on prostate cancer cell migration.

Loss of Synpo2 expression correlates with increased invasive tumor development, an observation seemingly at odds with the serum-stimulated, pro-migratory phenotype that results when Synpo2 expression is restored in PC3 cells. Synpo2 is expressed in both prostate epithelial and basal cells [7, 8], and can promote or inhibit cell migration in response to different external stimuli [16]. Thus, in response to the complex mixture of signaling molecules present in a tumor microenvironment, Synpo2 expression could inhibit migration of neoplastic epithelial cells or promote migration and cellular interactions of basal cells to maintain the integrity of the basal layer, either of which would inhibit tumor cell invasion.

Our present results provide a second intriguing possibility to explain the effects of Synpo2 on cancer cell invasion. Tumor cells are known to migrate by at least two distinct mechanisms in 3D tissues, mesenchymal motility or amoeboid cell migration. Mesenchymal migration involves actin-driven lamellipodial protrusions with FA formation and maturation, while amoeboid migration is less adhesive and is frequently accompanied by actin polymerization-independent membrane blebbing [51]. Disruption of actin cortex-membrane interaction and reduced substratum adhesion promote bleb-dependent migration, and the ability to alternate between these two migration phenotypes may enhance tumor cell migration and invasion. As we have now shown, Synpo2 alters actin dynamics near the membrane and enhances actin polymerization and FA formation, properties known to favor lamellipodia formation and inhibit bleb formation. The extensive formation and retraction of membrane blebs in Synpo2-deficient PC3 cells (Figure 1 and Supplementary Video 1) is also consistent with the concept that Synpo2 may tip the balance toward mesenchymal versus amoeboid migration. If so, then Synpo2 expression might inhibit tumor cell invasion in 3D environments by repressing bleb formation and amoeboid migration. Additional studies in both 2D and 3D environments of Synpo2 effects on membrane protrusions should provide further mechanistic insights into the function of this invasive cancer biomarker.

## MATERIALS AND METHODS

### Cells and reagents

PC3 cells were provided by David Hoskin (Dalhousie University) and were originally obtained in 2009 from the ATCC (ATCC® CRL-1435™). Cells were screened by RT-PCR and compared to benign prostate hyperplasia (BPH-1) cells to confirm they were hemizygous for Synpo2 [17], and were submitted to DDC Medical for cell line DNA typing, which confirmed their identity as PC3 cells. Cells were also routinely screened for mycoplasma and were grown in antibiotic-free medium, and remained free of microbial contamination throughout this study. Cells were cultured in high glucose Dulbecco's modified Eagle's medium (Invitrogen) supplemented with heat-inactivated 10% fetal bovine serum (FBS) (Invitrogen) at 37°C in a 5% CO_2_ atmosphere. Anti-cmyc (Sigma) and anti-phospho-paxillin (Cell Signaling) antibodies, Alexa 555-conjugated phalloidin (Molecular Probes), Alexa 488-conjugated anti-mouse and Alexa 633-conjugated anti-rabbit secondary antibodies (Molecular Probes), CK666 (EMD Millipore), and blebbistatin (Sigma) were purchased from the indicated commercial sources.

### Molecular cloning

All studies were performed using isoform Synpo2As (GenBank accession number CAZ66141), the 698-residue short isoform of Synpo2A also known as ΔN-MYO1 [17]. Synpo2As was N-terminally myc-tagged and cloned into the pBMN retroviral vector as described previously [16], and was also amplified using primers containing BamHI and SalI sites and subcloned into the BglII and SalI sites in plasmids peGFP or peYFP. PCR reaction mixtures were denatured at 95°C for 30 sec, annealed at 56°C for 30 sec, and extended at 72°C for 1 min/kb. These steps were repeated for 25 cycles followed by a 10 min final extension time at 72°C. The sequence of all clones was confirmed (McLab).

### Retroviral transduction system for stable Synpo2 expression

Synpo2 in pBMN was introduced into Phoenix retroviral packaging cells using PEI transfection reagent. After 48 h, supernatants were collected, cell debris was removed by passage through 0.45 μm low protein-binding filters, and Sequabrene (4 μg/ml; Sigma) was added to the viral supernatant. The virus-containing supernatant was immediately used for infection or stored in single-use aliquots at −80°C. Cells were infected with retroviruses carrying the indicated constructs for 24 h and cultured for another 24 h with fresh growth medium before selection with 1 μg/ml of puromycin (Invitrogen) for 3 days. Dead cells and debris were periodically removed by refeeding the monolayers with selection medium.

### Indirect immunofluorescence microscopy

PC3 cells (2 × 10^4^ cells) cultured overnight on tissue culture treated 15 mm glass coverslips (Fisher Scientific) were serum-synchronized by serum starvation for 24 h followed by addition of 10% serum to stimulate chemokinetic activity. At the indicated times post-serum stimulation, cells were fixed for 20 min at room temperature using 3.7% formaldehyde in phosphate-buffered saline (PBS), then permeabilized for 10 min at room temperature with 0.25% Triton X-100 in PBS. Fixed cells were blocked with 1% bovine serum albumin (BSA) in PBS, incubated overnight at 4°C with primary antibody dilutions (anti-myc [1:1000] or anti-phospho-paxillin [1:50]), extensively washed with blocking buffer, and bound primary antibody visualized using the indicated anti-mouse or anti-rabbit fluorophore-conjugated secondary antibodies (1:1000). Filamentous actin was visualized using a 1:40 dilution of Alexa555-conjugated phalloidin. Cells were mounted in Prolong Gold antifade reagent (Invitrogen) and immunofluorescent images were acquired on a Zeiss LSM 510 Meta laser scanning confocal microscope. Images were processed using Photoshop CS6 software (Adobe) using only linear adjustments.

### Stable shRNA knockdown of myosin regulatory light chain

Three shRNAs targeting the coding sequence of the myosin II regulatory light chain were designed using RNAi Central software (http://cancan.cshl.edu/RNAi_central/RNAi.cgi?type=shRNA). The shRNAs were individually cloned into the EcoRI and XhoI sites of plasmid pSMN (kindly provided by Craig McCormick). The shRNA constructs were transfected into PC3 cells to measure the knockdown efficiency, which was assessed by western blotting. The construct displaying the maximum knockdown efficiency was selected and used to generate shRNA retroviruses using the retroviral transduction system except that cells were selected with 1 mg/ml neomycin (Invitrogen) for 5 days. The sequence of the selected RLC shRNA was 5′-TGCTGTTGACAGTGAGCGCTTGCTTGC TTTGATGAAGAAGTAGTGAAGCCACAGATGTACT TCTTCATCAAAGCAAGCAAATGCCTACTGCCTCG GA-3. The same procedure was used to generate cells stably expressing an Open Biosystems non-targeting control shRNA (GE Healthcare Bio-Sciences)

### Live cell imaging analysis

PC3 cells stably expressing Lifeact-RFP (2 × 10^4^ cells) were cultured on 18 mm round glass coverslips (Fisher Scientific) overnight and transfected with either YFP- or GFP-tagged Synpo2 construct using Lipofectamine LTX. Cells were serum-starved overnight starting at 6 h post-transfection, stimulated with 10% FBS, and examined using a Marianas^TM^ spinning disk confocal microscope system (3i Intelligent Imaging Innovations), equipped for live cell analysis. Transfected PC3 cells were imaged every minute for 90 min within a temperature controlled (37°C) chamber and images were acquired with Slidebook (Version 5.0) imaging software. During image acquisition, cells were maintained in HBSS buffer (0.137 M NaCl, 5.4 mM KCl, 0.25 mM Na_2_HPO_4_, 0.44 mM KH_2_PO_4_, 1.3 mM CaCl_2_, 1.0 mM MgSO_4_, 4.2 mM NaHCO_3_) supplemented with filtered FBS. Blebbistatin at the indicated concentration was added to cells to inhibit myosin contractibility after actin bundles assembled in the cell body. For DIC imaging analysis, mock- or Synpo2-transduced cells were imaged at a 20x magnification for 2.5 h post-serum stimulation, and the migratory paths of 45 cells from three samples were used to calculate total displacement and endpoint displacement using Slidebook.

### Transwell cell migration assays

Transwell migration assays were performed as previously reported [16], using 24-well BD-Falcon transwell units with 8 μm porous polycarbonate membranes (VWR). The top chamber was seeded with 0.75 x10^5^ cells/well and both chambers were filled with DMEM plus 10% FBS. When indicated, 10 μM blebbistatin was added to the top and bottom chambers at the indicated concentrations. Cells were incubated for 24 h, and cells on the bottom of the membrane fixed with methanol, stained with DAPI for 10 min, and five random fields were imaged with a 20x objective, using duplicate or triplicate filters for each sample. The average number of cells/field that transmigrated through the membrane was quantified using ImageJ software.

### Statistical analysis

All results are expressed as the mean ± SEM (standard error of the mean). Statistical significance was assessed by a two-tailed Student's t-test.

## SUPPLEMENTARY MATERIAL FIGURES AND VIDEOS








