# Supplementary material for: Synaptopodin-2 induces assembly of peripheral actin bundles and immature focal adhesions to promote lamellipodia formation and prostate cancer cell migration
Source: Oncotarget. 2015 Mar 14;6(13):11162–74. doi: 10.18632/oncotarget.3578 (PMC4484447; doi:10.18632/oncotarget.3578)
Supplement: Supplementary file 1 [file oncotarget-06-11162-s001.pdf]

Synaptopodin-2 induces assembly of peripheral actin bundles and immature focal adhesions to promote lamellipodia formation and prostate cancer cell migration

Supplementary Material

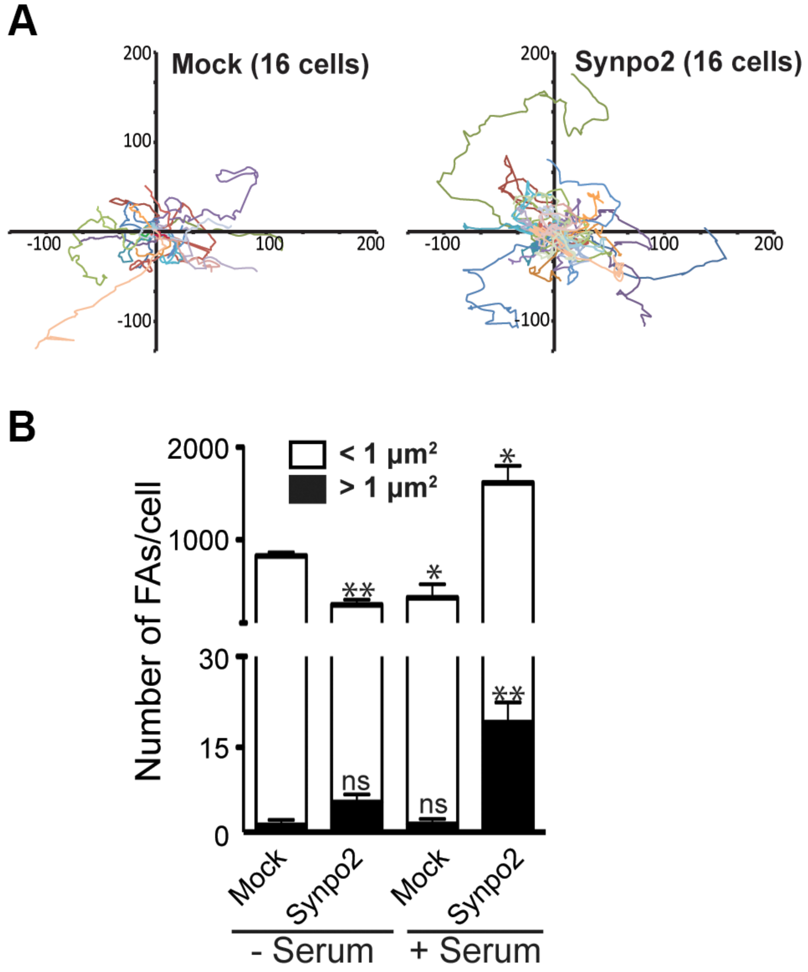

Supplementary Figure 1: (**A**) Cell migration of serum-synchronized mock- and Synpo2-transduced PC3 cells was imaged for 2.5 h using DIC video microscopy, and the migratory paths of 16 cells (colored lines) were plotted, shifting each track to start at the plot origin. Cell migration was assessed from Supplementary Video 1, and total displacement and endpoint displacement are presented in Figure 1B. (**B**) Quantification of immature and mature FAs in mock- and Synpo2-tranduced PC3 cells. Serum-synchronized mock- and Synpo2-expressing PC3 cells were fixed 1 h post-serum stimulation and immunostained with anti-phospho-paxillin

antibodies, and the numbers of FAs in the indicated size classes were quantified using ImageJ. Results are presented as the total number of FAs/cell in the indicated size classes ± SEM for 35 cells from random fields under each condition $**P<0.01$; $*P<0.05$ relative to the serum-starved mock-transduced cells; ns= not significant.

Supplementary Videos

Supplementary Video 1: Time-lapse images of mock- and Synpo2-transduced cells. Mock- or Synpo2-transduced cells were serum-synchronized and imaged by DIC at 20x magnification every 1.5 min for 2.5 h at 37°C, starting at 10 min post-serum stimulation. Scale bar=10 µm. Note the continual formation of large, sheet-like lamellipodia in Synpo2-expressing cells, and the extensive formation and retraction of circular membrane blebs in mock-transduced cells. Stills from this video are presented in Figure 1A.

Supplementary Video 2: Retrograde flow of Synpo2. Left: YFP-tagged Synpo2-transfected cells in the presence of serum were imaged by fluorescence microscopy at 40x magnification at 1 min intervals for 43 min. Right: time-lapse composite images of YFP-tagged Synpo2 to visualize retrograde flow of Synpo2. Green indicates the cumulative path of Synpo2 over time and red indicates each succeeding time point. Scale bar=10 µm.

Supplementary Video 3: Retrograde flow of actin and Synpo2. Cells stably expressing Lifeact-RFP and co-transfected with GFP-tagged Synpo2 were serum-synchronized and imaged by fluorescence microscopy at 40x magnification and 1 min intervals for 50 min. Left panel shows the GFP channel (Synpo2), middle panel shows the red channel (actin), and right panel the color merge. Note that cells not exhibiting detectible Synpo2 expression (bottom cell in center and right panels) exhibited no obvious inward flow of F-actin. In contrast, Synpo2-associated actin bundles near the cell periphery were observed flowing centripetally from the periphery to developing actin bundles in the cell body (top cell in all three panels).
